# Quinpramine Ameliorates Rat Experimental Autoimmune Neuritis and Redistributes MHC Class II Molecules

**DOI:** 10.1371/journal.pone.0021223

**Published:** 2011-06-16

**Authors:** Gerd Meyer zu Hörste, Anne K. Mausberg, Johanna I. Müller, Helmar C. Lehmann, Stefan Löber, Peter Gmeiner, Hans-Peter Hartung, Olaf Stüve, Carsten Korth, Bernd C. Kieseier

**Affiliations:** 1 Department of Neurology, Medical Faculty, Heinrich-Heine-University, Düsseldorf, Germany; 2 Department of Medicinal Chemistry, University of Erlangen-Nürnberg, Erlangen, Germany; 3 Department of Neurology, University of Texas Southwestern, Dallas, Texas, United States of America; 4 Neurology Section, Medical Service, VA North Texas Health Care System, Dallas, Texas, United States of America; 5 Institute for Neuropathology, Heinrich-Heine-University, Düsseldorf, Germany; University of Muenster, Germany

## Abstract

Activation of inflammatory cells is central to the pathogenesis of autoimmune demyelinating diseases of the peripheral nervous system. The novel chimeric compound quinpramine—generated from imipramine and quinacrine—redistributes cholesterol rich membrane domains to intracellular compartments. We studied the immunological and clinical effects of quinpramine in myelin homogenate induced Lewis rat experimental autoimmune neuritis (EAN), a model system for acute human inflammatory neuropathies, such as the Guillain-Barré syndrome. EAN animals develop paresis of all limbs due to autoimmune inflammation of peripheral nerves. Quinpramine treatment ameliorated clinical disease severity of EAN and infiltration of macrophages into peripheral nerves. It reduced expression of MHC class II molecules on antigen presenting cells and antigen specific T cell proliferation both *in vitro* and *in vivo*. Quinpramine exerted its anti-proliferatory effect on antigen presenting cells, but not on responder T cells. Our data suggest that quinpramine represents a candidate pharmaceutical for inflammatory neuropathies.

## Introduction

Acute inflammatory neuropathies are rare [1], but often severely disabling disorders of the peripheral nervous system and are associated with relevant morbidity and mortality [2]. Current therapeutic options remain incompletely effective [3].

The prototypic acute inflammatory neuropathy Guillain–Barré syndrome (GBS) most commonly manifests as ascending flaccid tetraparesis with less pronounced sensory deficits [4]. Its clinical course and long-term outcome are highly variable [2] and the prognosis is thought to be determined by the degree of axonal loss. Demyelinating and axonal variants can be distinguished [5]. In the demyelinating variants histopathology features multifocal mononuclear cell infiltration, demyelination and secondary axonal loss [6]. These pathological characteristics are recapitulated in experimental autoimmune neuritis (EAN), an animal model of GBS that can be induced in susceptible rodent strains by active immunization or passive transfer paradigms [7]. This animal model has enabled testing of multiple experimental therapies aimed at ameliorating GBS in a preclinical setting [8].

A promising novel compound for the possible future treatment of inflammatory disorders of the nervous system has recently been identified. In an *in vitro* screen for compounds inhibiting prion protein amplification the chimeric drug quinpramine – fused from the anti-depressant imipramine and the malaria treatment quinacrine – was highly effective [9]. Mechanistic studies suggested that the anti-prion effect is co-mediated by redistributing cholesterol to intracellular compartments [9]. The organization of antigen presenting molecules at the immunological synapse is associated with cholesterol distribution in cellular membrane microdomains [10], [11]. It was thus conceivable - though previously unknown - that quinpramine could also be effective in inflammatory diseases [12].

In experimental autoimmune encephalomyelitis (EAE) – the animal model of multiple sclerosis – quinpramine reduced clinical disease severity in both a preventive and therapeutic paradigm [13]. Quinpramine treatment also improved histopathological indicators of EAE severity. Splenocytes from quinpramine treated animals exhibited reduced antigen-specific T cell proliferation and secretion of the pro-inflammatory cytokines IFN-γ and IL-17 [13] indicating a reduced autoimmune activation due to quinpramine treatment, however, the precise mode of action remained elusive. Therefore, we assessed the clinical and immunological efficacy of quinpramine in EAN and studied the underlying mechanism of effect.

## Materials and Methods

### Induction of experimental autoimmune neuritis

EAN was induced as previously described [14]. Briefly, female Lewis rats (150–200 grams) aged 8–12 weeks (Charles River Laboratories) received subcutaneous injections (200 µl) in the hind footpad of 6–8 mg of bovine peripheral nerve myelin (BPNM) generated as previously described [15] emulsified in 100 µl PBS and mixed with 100 µl complete Freund's adjuvant (CFA, Difco) containing 1 mg/ml heat inactivated Mycobacterium tuberculosis (H37Ra). Severe and moderate EAN were induced with 8 and 6 mg of BPNM per animal, respectively. Four independent EAN treatment experiments were performed. All experiments contained at least three experimental groups: no EAN, EAN vehicle and EAN quinpramine therapeutic. In sum, 30 animals were analyzed per treatment group (4–10 animals per group per experiment). Two of the four total experiments additionally contained experimental groups for quinacrine and preventive quinpramine treatment (4–10 animals per group per experiment), respectively. Clinical scoring was performed work-daily by an observer blinded towards treatment (A.K.M.) and the score ranged from grade 0 to 10 (0—no signs to 10—death) as previously described [16]. Animal experimentation was approved by local state authorities (Landesamt für Natur, Umwelt und Verbraucherschutz Nordrhein-Westfalen) under the approval reference number 8.87-50.10.34.08.336.

### Quinpramine treatment

Quinpramine was synthesized as previously described [9], [17]. Animals were randomized to receive oral treatment with quinpramine solution by oral gavage by force feeding three times per week starting at day 2 after immunization (preventive) or starting at day 10 after immunization (therapeutic). Quinpramine was dissolved in 5% of total volume 100% ethanol and then diluted with 95% safflower oil. The drug-oil emulsion was prepared freshly every day and was adjusted to the animals' body weight respectively. Treatment (2 mg/200 µl solution/rat) was performed three times per week corresponding to a final dose of 6 mg/week/rat. Control animals were fed 5% ethanol/95% safflower oil mixture only. In an initial experiment, Quinpramine dosages between 6–15 mg/week/rat were tested, but did not further improve treatment responses, while eliciting no observable adverse effects (data not shown). Three subsequent independent quinpramine treatment experiments (n = 6–10 per treatment group) were performed using the 6 mg/week/rat dosage. In a control experiment, EAN rats (n = 6) received the quinpramine precursor substance quinacrine (2 mg/200 µl solution/rat) diluted in 5% ethanol/95% safflower oil mixture by force feeding three times per week. For *in vitro* application, quinpramine was dissolved in DMSO (1 mg/ml) and used at a final concentration of 50–200 nM for 1–24 hours in quadruplicate wells.

### Histology

At the peak of EAN severity (day 14 post immunization) a randomly chosen half of the treated group (n = 5) was sacrificed by CO_2_ narcosis and sciatic nerves were dissected and paraffin or epoxy resin embedded as previously described [18]. Paraffin sections (10 µm) were stained with haematoxylin/eosin and an anti-CD68 (ED1, Serotec) or anti-MHC-II antibody (MCA2687R, Serotec) using DAB based staining as previously described [19]. The entire nerve cross section was photographed using a conventional microscope (Axioplan 2, Carl Zeiss) and the digital photographs were photomerged using Adobe Photoshop CS3 software (Adobe Systems). The nerve cross sectional area occupied by mononuclear cell infiltrates was measured and the number of CD68 positive and MHC-II positive nuclei was counted using ImageJ software (v1.41, NIH). Semi thin (0.5 µm) section were cut from epoxy resin embedded sciatic nerves, stained with methylene-blue as previously described [18] and photographed.

### Filipin staining

The murine macrophage cell line RAW264.7 (ATCC) was cultured in the presence of quinpramine (200 nM) for six days before staining for cholesterol with filipin (25 µg/ml, Sigma) in 90% glycerol and 1.5 mg/ml glycin in PBS for 30 minutes at room temperature. Stained cells were photographed using a conventional microscope (Axioplan 2, Carl Zeiss).

### *Ex vivo* splenocyte proliferation

At day 14 post immunization spleens from randomly chosen quinpramine and vehicle treated EAN rats were dissected under sterile conditions and passed through a 40 µm cell strainer followed by ammonium chloride based erythrocyte lysis (BD Bioscience). Derived responder splenocytes (2×10^5^/well) were cultured in flat bottom 96-well plates in standard T cell medium (IMDM, 5% fetal calf serum, 2 mM L-glutamine, 50 µM β-mercaptoethanol, 100 U/ml Penicillin und 100 µg/ml Streptomycin) in the presence of varying concentrations of BPNM (0.1–100 µg/ml) in quadruplicate wells for 96 hours. ^3^H-Thymidin was added for the last 24 hours and cell proliferation was measured by detection of incorporated radioactivity as counts per minute.

### Ovalbumin peptide-specific T cell proliferation

Mouse splenocytes were prepared from OTII mice expressing a T cell receptor recognizing the MHC- II restricted Ovalbumin peptide amino acids 323–339 (Ova_323–339_) (OTII cells) (Jackson Laboratories, strain 004194). OTII cells were cryopreserved in 50% DMEM, 40% FCS and 10% DMSO and thawed immediately before usage. Cocultures were prepared using responder OTII cells and irradiated (1000 Gy) syngenic antigen presenting cells (APC) (1.5×10^5^, respectively) in the presence of Ova_323–339_ (10 µM, Peptides International) and proliferation was measured by ^3^H-Thymidin incorporation. Either APCs or responder cells were pre-incubated before coculturing with quinpramine at varying dosages (0–200 nM) for 12 hours. Combinations of differently pre-treated cells were tested for proliferation in quadruplicate wells and two independent experiments were performed.

### Schwann cell culture

Conditionally immortalized mouse Schwann cell cultures were maintained as previously described [20], [21]. Schwann cells were pre-treated with IFN-γ (10 ng/ml, R&D Systems) for 24 hours and incubated with quinpramine for further 1 to 24 hours at 1 to 500 nM. Schwann cells were stained for MHC class II (MHC-II, Serotec MCA2687R) using a DyLight649 labelled goat-anti-mouse secondary antibody (Serotec STAR74D649) and analyzed by flow cytometry.

### Flow Cytometry

For extracellular staining, splenocytes from quinpramine or vehicle treated rats were co-stained for cell surface CD11b/c (BD 554862) and MHC class II (MHC-II, Serotec MCA2687R) using a DyLight649 labelled goat-anti-mouse secondary antibody (Serotec STAR74D649). For intracellular staining cells were first stained with CD11b/c, fixed with 4% paraformaldehyde solution, permeabilized with PermBuffer and subsequently stained for intracellular MHC-II according to the manufacturers protocol (BD Biosciences). To analyze the MHC-II distribution *in vitro*, rat splenocytes of untreated and non-immunized rats were cultured in the presence of 100 U/ml rat recombinant interferon (IFN)-γ in the presence or absence of quinpramine (100 nM). The percentage of MHC-II^+^ cells and the fluorescence intensity of MHC-II staining were assessed. Three independent experiments with quadruplicate wells were performed. To assess the fluorescence pattern of quinpramine and its precursor substances, splenocytes were cultured under routine conditions for 1–6 hours in the presence of quinpramine (100 nM), quinacrine (100 nM), imipramine (100 nM) or a mixture of the later two (each at 100 nM). Cells were washed three times with PBS and analyzed for fluorescence in the channels FITC, PE, PerCP-Cy5.5, PE-Cy7, APC, APC-Cy7, PacBlue, AmCyan. All flow cytometry was performed using a FACSCanto II flow cytometer. Two independent experiments with triplicate wells were performed. All flow cytometry data were analyzed using FlowJo software (v7.2.5 TreeStar).

### Data analysis

Data were statistically analyzed using GraphPadPrism 5.0 (GraphPad Software). The Wilcoxon-Mann-Whitney was used to test for statistically significant differences in clinical score values. Student's t-test for unrelated samples was used to test for statistically significant differences in all other analyses. Differences were considered significant at p-values<0.05.

## Results

### Quinpramine ameliorates clinical and histological manifestation of EAN

**Figure 1:**
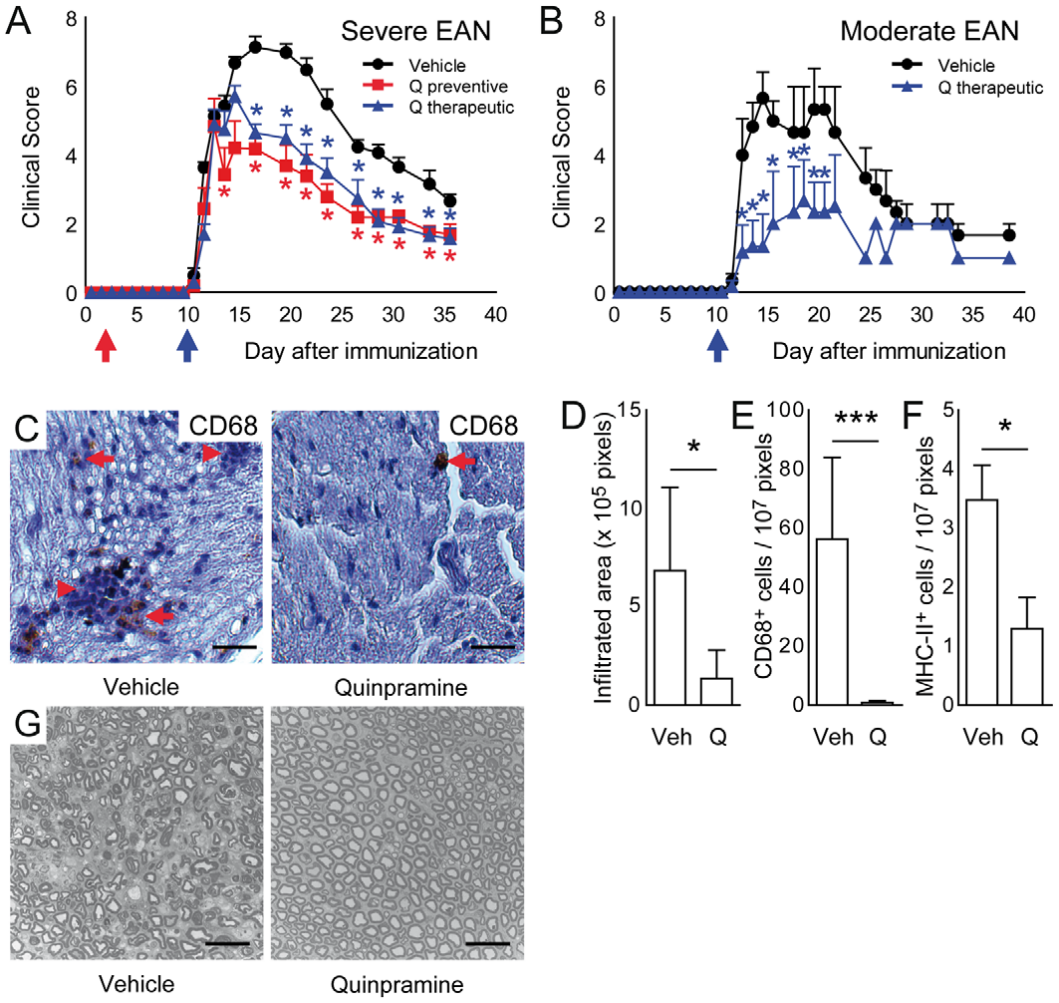
Quinpramine ameliorates clinical and histological features of EAN. (A) Severe EAN was induced in female Lewis rats by subcutaneous immunization with bovine peripheral nerve myelin homogenisates (BPNM, 8 mg/animal). Animals were force fed by oral gavage with quinpramine (2 mg/kg/week) three times per week from day 2 (Q preventive) or day 10 (Q therapeutic) after immunization or with vehicle alone (n = 10 per group). Clinical score ranging from 0 (healthy) to 10 (death) was assigned daily in a blinded fashion. Both preventive (grey squares) and therapeutic (black dotted line) quinpramine treatment in comparison to vehicle alone (black points) significantly reduced maximum EAN severity and disability. (B) Animals with moderate EAN (n = 6 per group) received oral vehicle or quinpramine (2 mg/kg/week) treatment starting at day 10 after immunization (Q therapeutic). Quinpramine treatment (blue triangles) significantly reduced peak severity of moderate EAN in comparison to vehicle treatment (black points). Plots represent mean ± SEM. (C) Sciatic nerves from randomly selected vehicle (left panel) and quinpramine (right panel) treated EAN rats were dissected at day 14 after immunization and stained against CD68. Peripheral nerves from vehicle treated EAN rats (left panel) exhibited multiple mononuclear cell infiltrates (red arrowheads). CD68^+^ macrophages were found in infiltrates and diffusely distributed within the endoneurium (red arrows). PNS from quinpramine treated EAN rats (right panel) did not contain relevant cellular infiltrates and only occasional CD68^+^ cells (red arrow). Scale bars represent 25 µm. (D–F) The endoneurial area occupied by inflammatory infiltrates (D) and the endoneurial density of CD68^+^ (E) and MHC-II^+^ cells (F) were significantly reduced in quinpramine treated animals. Plots represent mean ± SEM. (G) Epoxy resin embedded sciatic nerves of quinpramine treated EAN rats at day 14 after immunization were cut to semi thin (0.5 µm) sections. Nerves from vehicle treated EAN rats exhibited mononuclear cell infiltration, myelin degradation and endoneurial oedema (left panel), while the PNS of quinpramine treated EAN rats did not contain relevant infiltrates and myelin was well maintained (right panel). Scale bars represent 20 µm. * p<0.05, *** p<0.005.

We first tested whether quinpramine – known to reduce inflammation and clinical manifestation of EAE – would be effective in inflammatory peripheral neuropathies. Severe EAN was induced in female Lewis rats and animals received oral quinpramine treatment by oral gavage by force feeding three times per week. Quinpramine significantly reduced EAN peak severity both when initiated early at day 2 after immunization (preventive paradigm) (Fig. 1A) and when starting at day 10 after immunization at the onset of first clinical signs of EAN (therapeutic paradigm) (Fig. 1A). In an independent experiment we tested the potential of quinpramine to ameliorate EAN with moderate peak severity equivalent to mild GBS. Here, therapeutic quinpramine also significantly ameliorated EAN (Fig. 1B). Moderate PNS inflammation may be easier to influence therapeutically. We did not observe phenotypical signs of toxicity in quinpramine treated animals. Preventive treatment with the quinpramine precursor substance *quinacrine* did not ameliorate clinical signs of EAN (Fig. S1), suggesting a quinpramine specific effect. Subsequent *ex vivo* analyses were restricted to samples from severe EAN experiments. In sciatic nerve paraffin sections of vehicle treated, but not in quinpramine treated animals we found widespread infiltration of mononuclear cells (Fig. 1C). We found CD68 positive cells distributed both diffusely and within mononuclear infiltrates at lower frequencies in quinpramine treated animals (Fig. 1C). The nerve cross sectional area occupied by mononuclear infiltrates was significantly, approximately 5-fold reduced after quinpramine treatment (Fig. 1D). The density of CD68 positive (Fig. 1E) and of MHC-II positive (Fig. 1F) cells was significantly reduced in quinpramine treated animals. In peripheral nerve semi-thin sections we found preserved myelin integrity and also reduced cellular infiltrates in quinpramine treated animals (Fig. 1G). This indicates that quinpramine ameliorates EAN by reducing peripheral nerve inflammation and reducing subsequent myelin destruction.

### Quinpramine reduces cell surface presence of MHC class II on APCs *in vivo*

**Figure 2:**
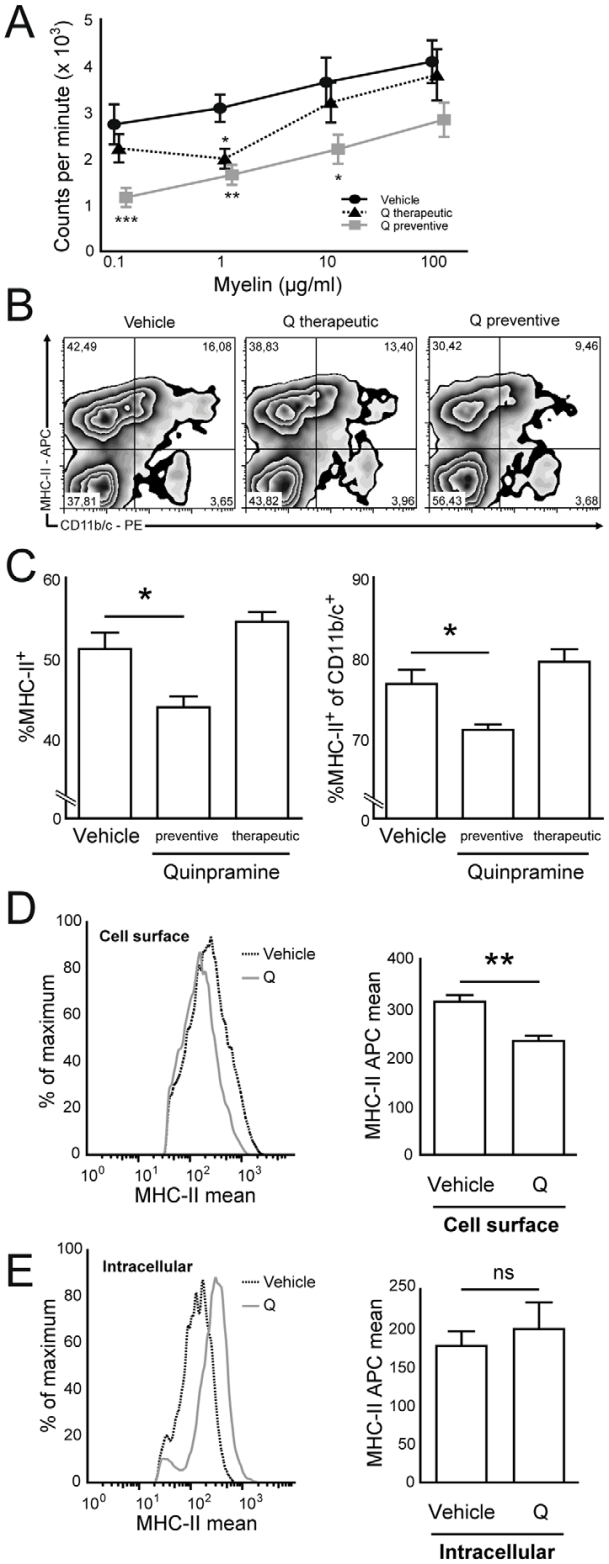
Quinpramine reduces antigen specific T cell proliferation and cell surface MHC class II availability *in vivo*. (A) Spleen cell suspensions were generated from rats at peak EAN severity (d14 post immunization) treated with vehicle (n = 4) or treated with quinpramine from day 2 (Q preventive, n = 4) or from day 10 after immunization (Q therapeutic, n = 4). Splenocytes were restimulated with increasing concentrations of bovine peripheral nerve myelin (BPNM) homogenate (0.1–100 µg/ml). Proliferation, as measured by ^3^H-thymidine incorporation, was significantly reduced after preventive quinpramine treatment (blue triangles) in comparison to vehicle treatment (black dots). Splenocytes from therapeutically treated animals (red squares) exhibited a partially significant trend at low antigen concentrations towards reduced proliferation. (B) Splenocytes from vehicle and quinpramine treated rats at peak EAN severity were analyzed for extracellular MHC-II and CD11b/c expression and representative flow cytometry contour plots are depicted. The percentage of total MHC-II^+^ cells (top quadrants) and the percentage of MHC-II^+^ cells of all CD11b/c^+^ cells (top right quadrant) were reduced after preventive quinpramine treatment *in vivo*. (C) The average proportion of MHC-II positive splenocytes (left panel) and the average percentage of MHC-II positive cells of all CD11b/c^+^ splenocytes (right panel) were significantly lower in animals having received preventive quinpramine treatment (middle bars, mean ± SEM) (n = 6 per group). (D) Splenocytes were stained for cell surface MHC-II and analyzed for mean fluorescence intensity. Splenocytes derived from quinpramine treated animals (Q, grey line) in comparison to controls (dotted black line) had reduced cell surface MHC-II expression in representative flow cytometry histograms (left panel) and on average (right panel). (E) Splenocytes from quinpramine treated animals (Q, grey line) exhibited an unchanged total extra- and intracellular MHC-II expression in comparison to controls (dotted black line) in representative (left panel) and average (right panel) analyses. The total MHC-II staining trend towards increase was non-significant. * p<0.05, *** p<0.005, ns not significant. All plots represent mean ± SEM.

To approach the possible mechanism underlying quinpramine therapy, we restimulated splenocytes from immunized rats with myelin homogenisates. Splenocytes from rats treated with quinpramine under a preventive paradigm had significantly lower proliferatory responses (Fig. 2A). Proliferation also had a tendency towards reduction in animals receiving therapeutic quinpramine, that was significant at one of the four antigen concentrations tested (1 µg/ml) (Fig. 2A). Given previous data that quinpramine redistributes cholesterol raft associated proteins, we hypothesized that quinpramine could reduce immune activation in EAN by reducing cell surface availability of MHC class II molecules. To experimentally test this hypothesis we compared the MHC-II expression on *ex vivo* isolated splenocytes between the three cohorts. Splenocytes from quinpramine treated animals had lower percentages of MHC-II^+^ cells in total and of CD11b/c^+^ APCs (Fig. 2B). On average, we found a significant reduction in the percentage of total MHC-II^+^ cells and in the proportion of MHC-II^+^ cells of CD11b/c^+^ cells after preventive quinpramine treatment (Fig. 2C). No difference was observed after therapeutic quinpramine treatment. To differentiate between reduced MHC-II expression and intracellular redistribution, we stained for extracellular and total MHC-II expression. Cell surface expression of MHC-II was significantly reduced in splenocytes from quinpramine treated animals (Fig. 2D). Permeabilized cells stained for total MHC-II exhibited an unchanged MHC-II expression (Fig. 2E). The trend towards an even increased expression was statistically not significant (Fig. 2E). This suggests that quinpramine reduces cell surface availability of MHC-II - possibly by intracellular redistribution - but not the overall MHC-II expression.

### Quinpramine reduces antigen specific proliferation responses by reducing cell surface MHC class II

**Figure 3:**
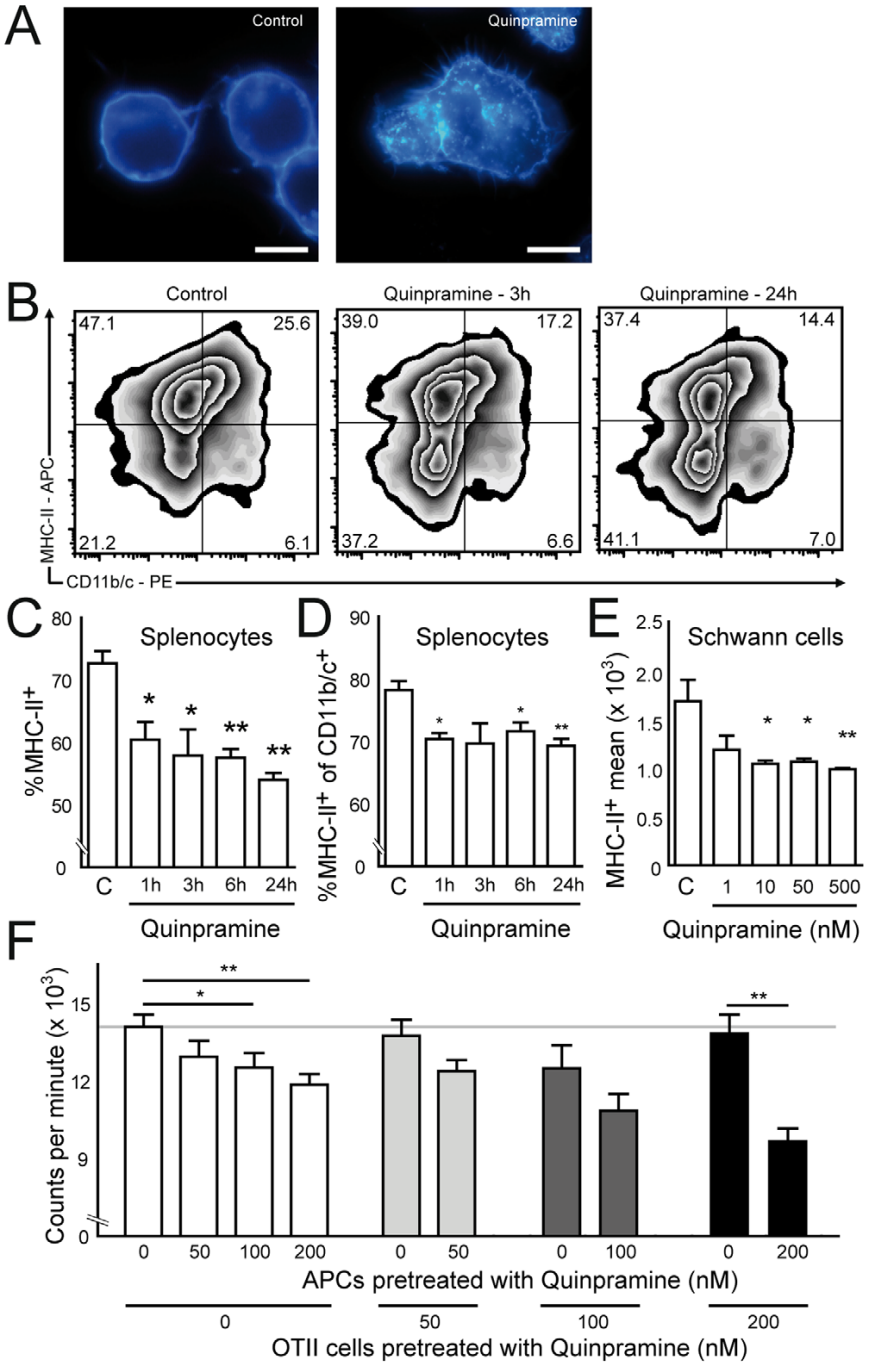
*In vitro* quinpramine reduces antigen specific T cells proliferation and reduces MHC class II expression on APCs. (A) A murine macrophage cell line was cultured in the absence (left panel) or presence of quinpramine (200 nM, right panel) for six days before labelling cholesterol with filipin. (B) Rat splenocytes were prestimulated with IFN-γ and incubated with or without quinpramine and analyzed for extracellular MHC-II and CD11b/c expression by flow cytometry (representative contour plots). In comparison to control cells (left panel), cells treated with quinpramine (100 nM) for 3 hours (middle panel) and 24 hours (right panel) show reduced percentages of MHC-II^+^ cells (top quadrants). (C) On average the percentage of total MHC-II^+^ cells was significantly reduced after 1 to 24 hours of quinpramine treatment (100 nM) in a time dependent manner. (D) The proportion of MHC-II^+^ cells of all CD11b/c^+^ cells was significantly reduced after quinpramine treatment (100 nM). Reduction did not change over time. * p<0.05, ** p<0.01 in comparison to controls. One representative out of four independent experiments is shown in A-D. (E) Conditionally immortalized mouse Schwann cells incubated with quinpramine for 24 hours show dosage dependent reduction in average MHC-II staining intensity. C control. (F) Irradiated splenocytes serving as antigen presenting cells (APCs) and responder splenocytes were generated from Ova_323–339_ T cell receptor transgenic OTII mice (OTII cells) and separately incubated with increasing concentrations of quinpramine (from 0 to 200 nM) for 24 hours. Combinations of cells pretreated with varying quinpramine concentrations (0–200 nM) were cocultured in the presence of 1 µg/ml Ova_323–339_ for 72 hours and proliferation was measured by 3H-Thymidin incorporation for the last 24 hours. Average values from three independent experiments are depicted. All non-indicated differences are statistically not significant. All plots represent mean ± SEM.

Extending previous reports, we studied the quinpramine effect on intracellular cholesterol distribution. Quinpramine treated macrophage cells had a tendency towards higher intracellular cholesterol content than untreated cells as visualized by filipin staining (Fig. 3A). In primary rat splenocytes quinpramine reduced cell surface expression of MHC-II in all splenocytes and in CD11b/c^+^ APCs (Fig. 3B). In time course analyses, the reduction of MHC-II expression was already significant after one hour and increased with longer quinpramine exposure (Fig. 3C). In CD11b/c^+^ cells, the reduction of MHC-II expression was not time dependent, but also statistically significant (Fig. 3D). Thus, quinpramine reduced MHC-II cell surface expression on APCs both *in vitro* and *in vivo*. MHC-II expression by local non-professional APCs can be relevant in inflammatory neuropathies [19], [20]. Cell surface MHC-II expression on IFN-γ prestimulated cultured murine Schwann cells was reduced in a concentration dependent manner (Fig. 3E). At the same time we observed that cellular quinpramine uptake was well traceable by its green fluorescence. We therefore used the fluorescence pattern of quinpramine and its precursor substances to trace its uptake. The pattern of fluorescence in quinpramine treated splenocytes differed from the pattern returned from cells incubated with either imipramine or quinacrine or a mixture of both (Fig. S2). This indicates that quinpramine is taken up into the cells, but is not cleaved into its precursor substances. To further assess the functional relevance of MHC-II redistribution in APCs we analyzed its target cell type using dissociated APC – T cell cocultures from Ovalbumin specific T cell receptor transgenic mice (Fig. 3G). Pre-treatment of APCs with quinpramine significantly reduced antigen specific T cell proliferation, while pre-treatment of only responder T cells did not affect proliferation (Fig. 3F).

## Discussion

We here demonstrate that quinpramine – a chimeric compound derived from quinacrine and imipramine – reduces peripheral nerve myelin specific autoimmune reactivity, peripheral nerve inflammation, demyelination and clinical impairments in a rat model of acute GBS. We further demonstrate that quinpramine reduces the cell surface presence, but not expression, of MHC-II molecules both *in vitro* and *in vivo* and therefore acts on APCs but not on responder T cells. We hypothesize that a diminished activation of myelin reactive T cells due to reduced MHC-II present on APCs underlies the clinical effectiveness of the drug. Especially since quinpramine is also clinically effective in our therapeutic paradigm, this novel anti-inflammatory drug may offer a future therapeutic option in inflammatory neuropathies. We found the effect to be quinpramine specific and not mirrored by its precursor substance quinacrine.

Quinpramine inhibits prion protein amplification [9] by altered intracellular cholesterol distribution and changed composition of cholesterol rich membrane domains – cholesterol rafts [9], [22]. The organization of antigen presenting and recognizing molecules at the immunological synapse is an at least partially raft mediated phenomenon [10], [11] and requires a well orchestrated interplay of antigen presenting and recognizing cells [23]. Therefore, a compound redistributing cholesterol rich rafts to intracellular compartments in a prion protein model could possibly also influence immunological responses [12]. We here found indirect evidence for such a mechanism: Quinpramine exerted its effect on APCs, but not on responder T cells. By morphology, intracellular cholesterol content appeared increased and by flow cytometry, MHC-II molecules were shifted from cell surface to intracellular compartments while total expression was unchanged. Although other mechanisms cannot be excluded, our data suggest that such an intracellular redistribution of MHC-II molecules may occur. Our data well explain how preventive quinpramine ameliorates EAN severity. Efficacy in the therapeutic treatment paradigm is more surprising, however, given the incomplete reduction of antigen specific proliferation (Fig. 2A) and unchanged percentage of MHC-II^+^ splenocytes (Fig. 2B, C). We speculate that two applications of quinpramine are sufficient for a clinical benefit in the therapeutic paradigm, but may not be sufficient to make the alterations traceable in our analyses. The *ex vivo* analyses (Fig. 2) mirror one time-point at day 14 post immunization (i.e. after two days of treatment), while the clinical score reflects the cumulative effect of more than 20 days of quinpramine treatment. This may explain the discrepancy.

Quinpramine thus represents a candidate pharmaceutical to possibly test in a clinical setting. In our therapeutic paradigm already two applications of quinpramine (i.e. on day 10 and 12 after immunization) were sufficient to reduce peak EAN severity on day 14 (Fig. 1A, B) suggesting a clinically fast and pronounced effect. It is remarkable that quinpramine was thus effective even after the priming phase in EAN is completed despite acting on APCs. One may speculate that continued activation during the effector stage of EAN remains relevant. Reduced MHC-II expression on Schwann cells indicates that quinpramine may reduce local T cell reactivation by conditional APCs. In our moderate EAN experiment quinpramine reduced peak disease severity more efficiently than in the severe EAN trial. Moderate EAN may be easier to therapeutically modify. From a clinical point of view quinpramine's oral bioavailability and relatively infrequent application are advantageous. We did not observe any obvious toxicity, but the exact safety profile of quinpramine remains unknown. Although we provide evidence that quinpramine is not cleaved to its precursor substances after intracellular uptake, one may speculate if quinpramine shares potential adverse effects with its precursor substances (reviewed in [12]). Any application of quinpramine to human subjects will require extensive preceding safety testing.

Given that numerous compounds are effective in EAN, but none of these has reached the bench-to-bedside transfer [8], testing yet another compound may be critically questioned. Other neuroinflammatory disorders, however, indicate that such a transfer is possible [24], [25] and the principal need for more effective therapies is undoubted in GBS [3].

In conclusion, our data suggest that quinpramine represents a possible candidate pharmaceutical to further establish in acute inflammatory neuropathies and that quinpramine takes effect on MHC class II expression on APCs. Such a mechanism may also be effective in other autoimmune disorders and may extend our therapeutic armentarium in autoimmune mediated disorders.

## Supporting Information

Figure S1 Quinacrine treatment does not ameliorate EAN. Moderate EAN was induced in female Lewis rats (n = 6 per group), who received oral vehicle or quinacrine (2 mg/kg/week) treatment – one of the precursor substances of quinpramine – starting at day 2 after immunization. Quinacrine treatment (green inverted triangles) did not significantly alter severity or course of moderate EAN in comparison to vehicle treatment (black points). Plots represent mean ± SEM.
(TIF)

Figure S2 Quinpramine treated cells exhibit a specific pattern of fluorescence. (A) Cultured rat splenocytes were left untreated (red line) or incubated for six hours with quinpramine (100 nM, green line) or a mixture of quinacrine and imipramine (each 100 nM, blue line) and analyzed by multicolor flow cytometry. Flow cytometry histograms depict that both quinpramine and mixture treated cells returned green fluorescence in the FITC (left panel) and AmCyan (middle panel) channel. (B) Average fluorescence intensities were calculated for each channel and quinpramine (green bars), mixture of quinacrine and imipramine (blue bars) and quinacrine only (yellow bars) all returned specific patterns of fluorescence in the FITC (left panel) and AmCyan (middle panel) channel. Imipramine (white bars) did not generate fluorescence in comparison to untreated controls (red bars). Notably, a mixture of quinacrine and imipramine did not return the same fluorescence intensity as quinpramine alone indicating that quinpramine is not cleaved to its precursors within the cells.
(TIF)
