# Supplementary figures and images for: Quinpramine Ameliorates Rat Experimental Autoimmune Neuritis and Redistributes MHC Class II Molecules
Source: PLoS One. 2011 Jun 16;6(6):e21223. doi: 10.1371/journal.pone.0021223 (PMC3116892; doi:10.1371/journal.pone.0021223)

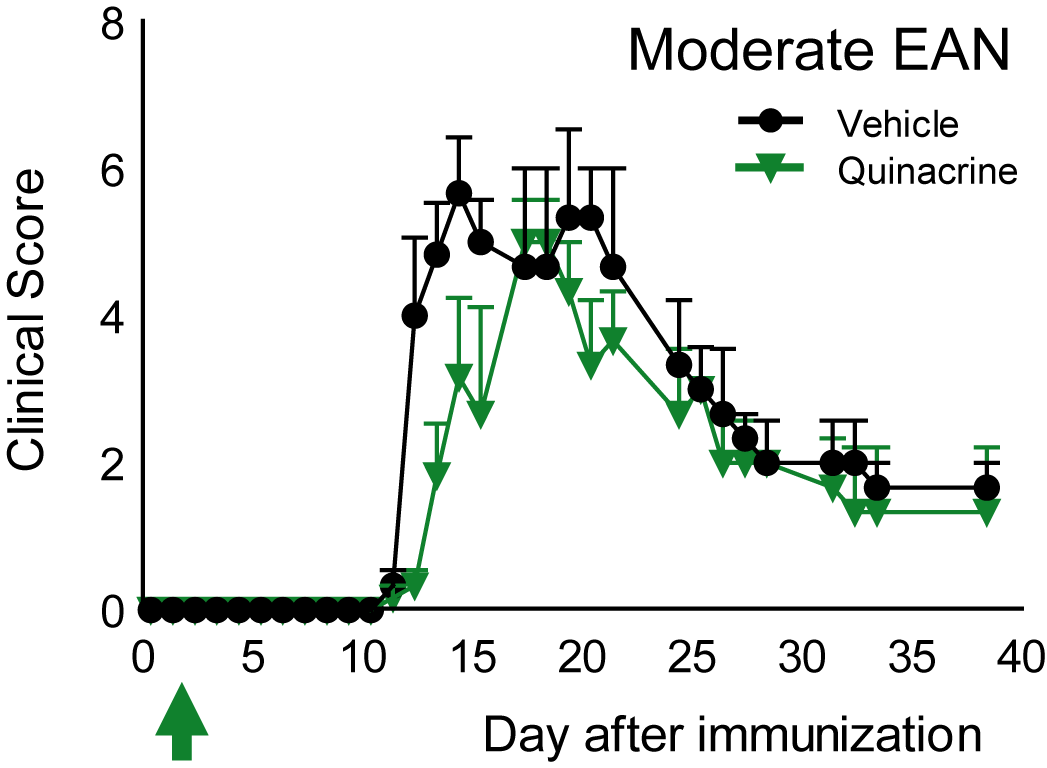

Supplement: Figure S1 — Quinacrine treatment does not ameliorate EAN. Moderate EAN was induced in female Lewis rats (n = 6 per group), who received oral vehicle or quinacrine (2 mg/kg/week) treatment – one of the precursor substances of quinpramine – starting at day 2 after immunization. Quinacrine treatment (green inverted triangles) did not significantly alter severity or course of moderate EAN in comparison to vehicle treatment (black points). Plots represent mean ± SEM. (TIF) [file pone.0021223.s001.tif]

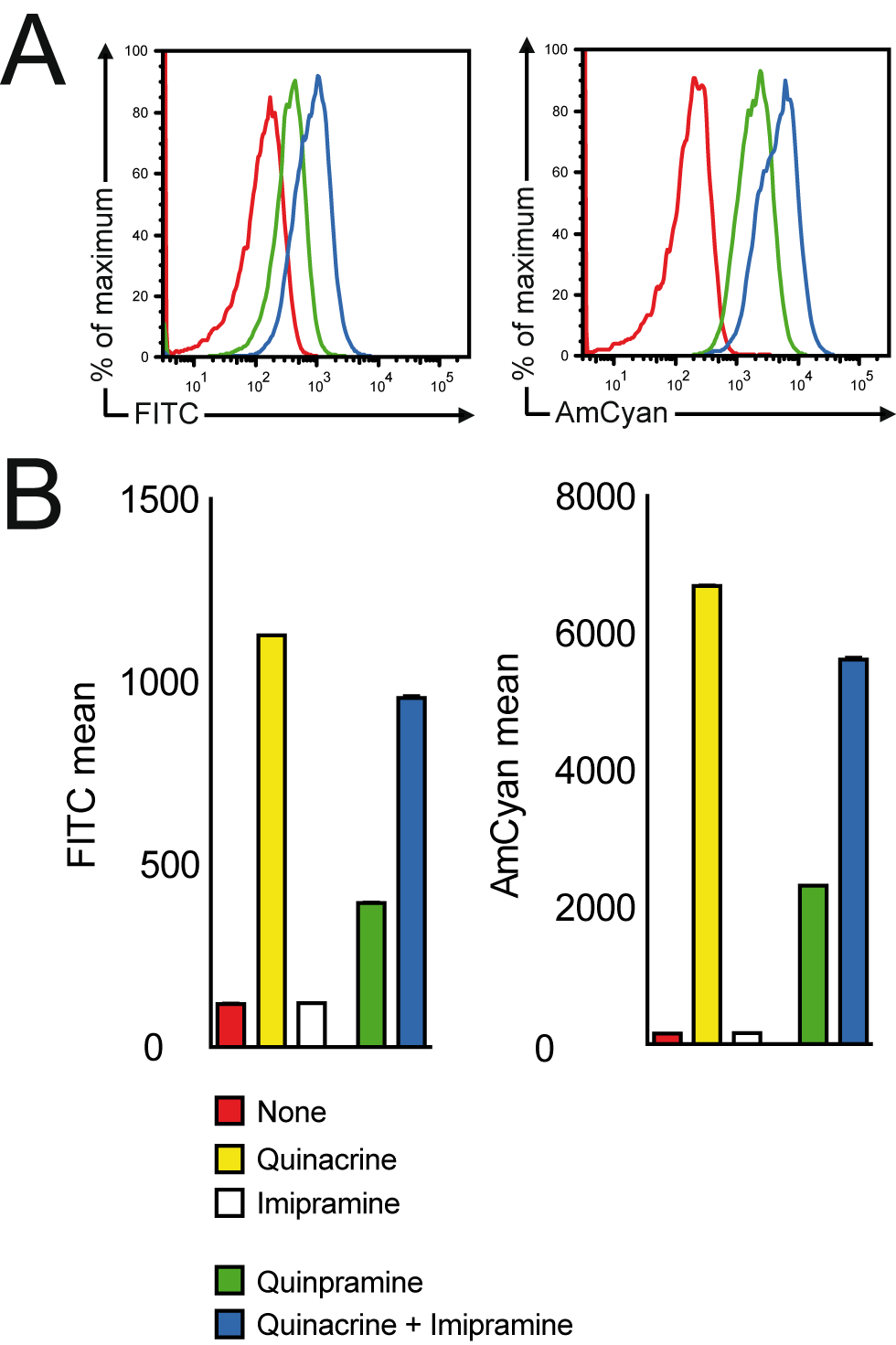

Supplement: Figure S2 — Quinpramine treated cells exhibit a specific pattern of fluorescence. (A) Cultured rat splenocytes were left untreated (red line) or incubated for six hours with quinpramine (100 nM, green line) or a mixture of quinacrine and imipramine (each 100 nM, blue line) and analyzed by multicolor flow cytometry. Flow cytometry histograms depict that both quinpramine and mixture treated cells returned green fluorescence in the FITC (left panel) and AmCyan (middle panel) channel. (B) Average fluorescence intensities were calculated for each channel and quinpramine (green bars), mixture of quinacrine and imipramine (blue bars) and quinacrine only (yellow bars) all returned specific patterns of fluorescence in the FITC (left panel) and AmCyan (middle panel) channel. Imipramine (white bars) did not generate fluorescence in comparison to untreated controls (red bars). Notably, a mixture of quinacrine and imipramine did not return the same fluorescence intensity as quinpramine alone indicating that quinpramine is not cleaved to its precursors within the cells. (TIF) [file pone.0021223.s002.tif]
